# Baseline Neutrophil-to-Lymphocyte Ratio and Two-Year Incident Distant Metastasis in Stage I–III Lung Cancer: A Single-Center Retrospective Cohort Study

**DOI:** 10.3390/jcm15166207

**Published:** 2026-08-11

**Authors:** Yuntao Wang, Jihong Zhang, Xiaopeng Liu, Xibing Zhuang, Qi Zhang

**Affiliations:** 1 Department of General Practice, Jinshan Hospital, Fudan University, Shanghai 201508, China; wangyuntao666@yeah.net; 2 Research Center for Clinical Medicine, Jinshan Hospital, Fudan University, Shanghai 201508, China; 3 Department of Respiratory Medicine, Jinshan Hospital, Fudan University, Shanghai 201508, China; 4 Department of Oncology, Jinshan Hospital, Fudan University, Shanghai 201508, China

**Keywords:** lung cancer, neutrophil-to-lymphocyte ratio, distant metastasis, stage I–III, retrospective cohort

## Abstract

**Background**: Distant metastatic relapse remains a major problem after treatment for lung cancer. We examined the association between pre-treatment neutrophil-to-lymphocyte ratio (NLR) and newly documented distant metastasis within 2 years among patients without distant metastasis at baseline. **Methods**: We retrospectively screened 527 consecutive unique patients with a hospital diagnosis of lung cancer who were managed between 1 January and 31 December 2022; follow-up continued for 24 months after initial treatment (administrative cutoff, 31 December 2024). After hierarchical exclusions, 186 complete cases remained. The primary analysis included 148 patients with stage I–III disease and used record-based incident distant metastasis within 24 months, determined by comprehensive clinical assessment. Logistic regression estimated the association between continuous NLR and the endpoint before and after adjustment for age, sex, and clinical stage. Stage IV patients were retained only in a secondary analysis of the original heterogeneous composite endpoint. **Results**: Thirty-one out of 148 patients with stage I–III disease (20.9%) developed distant metastasis within 2 years. Mean pre-treatment NLR was 2.83 in patients with an event and 2.07 in those without an event. Each one-unit increase in NLR was associated with higher odds of distant metastasis in the unadjusted model (odds ratio [OR] 2.71, 95% confidence interval [CI] 1.65–4.45; *p* < 0.001) and after adjustment for age, sex, and stage (adjusted OR 2.70, 95% CI 1.60–4.57; *p* < 0.001). In the secondary full-cohort analysis, 49 of 186 patients met the heterogeneous composite endpoint. **Conclusions**: Higher pre-treatment NLR was associated with 2-year incident distant metastasis in this stage I–III cohort. The result does not establish causality or a clinically actionable cut point and requires prospective external validation.

## 1. Introduction

Lung cancer remains a leading cause of cancer morbidity and mortality worldwide [1,2]. Distant relapse is a major determinant of outcome, yet patients with similar clinicopathologic features can follow markedly different metastatic courses after treatment, reflecting heterogeneity in tumor biology and the formation of premetastatic niches [3,4,5,6]. Readily available blood-based markers that help describe this risk therefore remain of clinical interest.

The neutrophil-to-lymphocyte ratio (NLR) is calculated from routine peripheral blood counts and reflects the balance between systemic inflammation and lymphocyte-mediated immune activity [7,8]. Neutrophils can support tumor progression through inflammatory signaling, immunosuppression, and the release of neutrophil extracellular traps, whereas lymphocytes contribute to antitumor surveillance [9,10,11,12,13]. Through these mechanisms, neutrophil-lineage cells help shape the metastatic niche and the tumor immune microenvironment [14,15], and the density and phenotype of tumor-infiltrating lymphocytes provide an accessible readout of antitumor immunity and treatment response [16,17,18].

Elevated NLR has been associated with poor outcomes in several lung cancer settings, including early-stage disease [19], immunotherapy-treated disease [20,21], and postoperative recurrence [22].

The original analysis combined incident distant metastasis in patients with stage I–III disease and metastatic status or progression in patients who had stage IV disease at baseline. These endpoints are not clinically equivalent. We therefore defined the stage I–III cohort as the primary analysis population and used a reduced adjustment model suited to the limited number of events. The stage-IV-inclusive result is reported only as a secondary analysis of the original composite endpoint.

## 2. Materials and Methods

### 2.1. Study Design and Participants

This single-center retrospective cohort study screened consecutive unique patients with a hospital diagnosis of lung cancer who received clinical management at the Department of Oncology, Jinshan Hospital, Fudan University, between 1 January and 31 December 2022. Repeated visits or admissions belonging to the same patient were consolidated using the unique hospital patient identifier before eligibility assessment. The index date was the date on which initial antitumor treatment was started at our institution. Each patient was assessed for 24 months after the index date, and the administrative end of follow-up was 31 December 2024. Medical-record extraction and research analyses were conducted after final ethical approval.

Eligibility required pathological confirmation of lung cancer, initial antitumor treatment at our institution, an available complete blood count obtained before initial treatment for calculation of NLR, complete baseline clinical and tumor-stage information, and sufficient medical-record documentation to determine the 24-month outcome. Patients with a documented metastatic event before 24 months were classified as outcome-positive. Classification as event-free required outcome documentation through 24 months; patients who died or were lost to follow-up before 24 months without a previously documented metastatic event were excluded as having insufficient follow-up.

**Figure 1:**
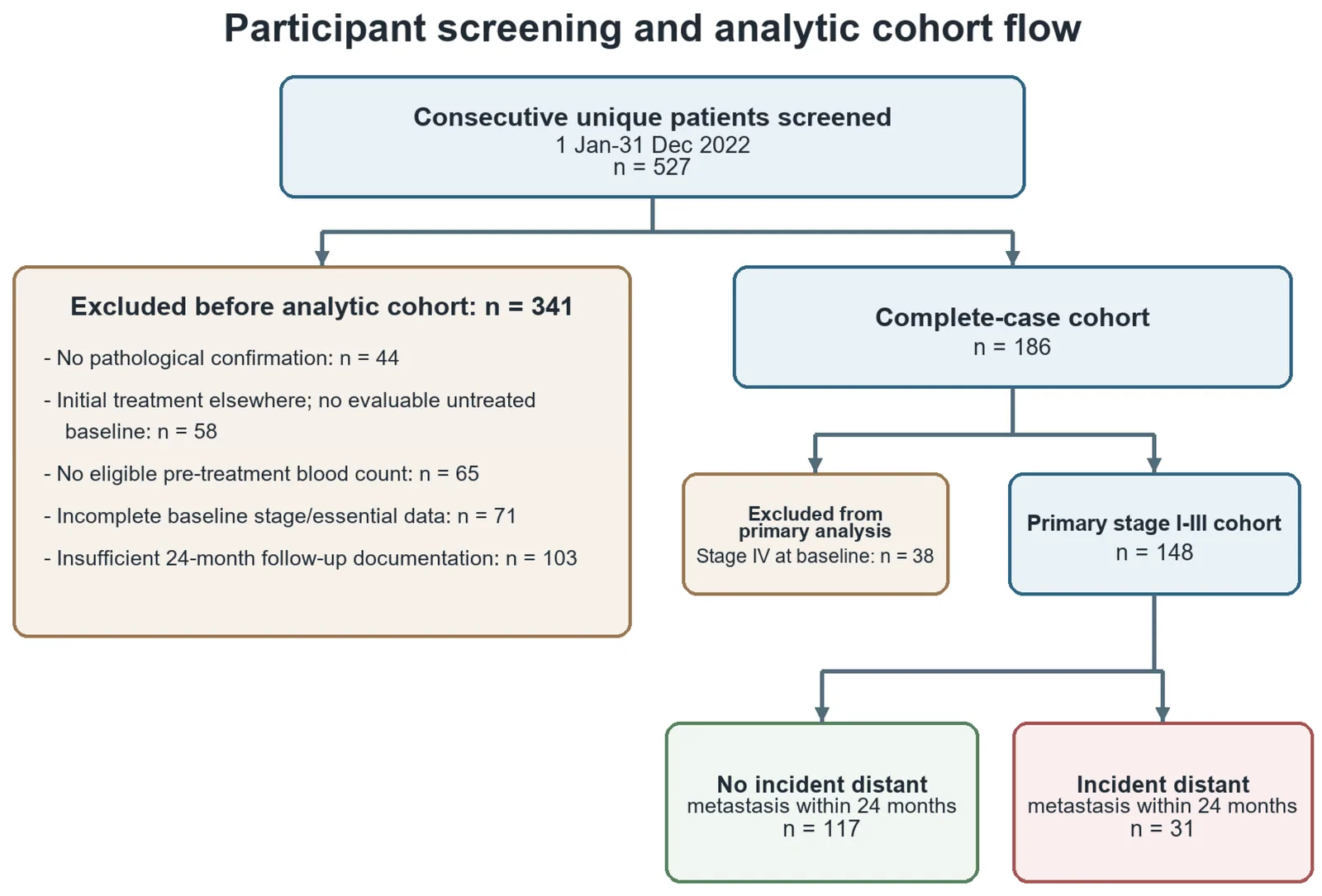
Participant screening and analytic cohort flow. Exclusion criteria were applied hierarchically so that each patient was assigned to one primary reason. Event-free classification required complete 24-month outcome documentation.

Exclusion criteria were applied hierarchically so that each patient was assigned to one primary exclusion category. Among 527 screened patients, 44 lacked pathological confirmation, 58 had initiated treatment elsewhere and had no evaluable untreated baseline, 65 lacked an eligible pre-treatment complete blood count, 71 had incomplete baseline stage or other essential clinical information, and 103 had insufficient 24-month follow-up documentation. The resulting complete-case cohort comprised 186 patients, and no imputation was performed. Thirty-eight patients with stage IV disease at baseline were excluded from the primary incident-metastasis analysis and retained only in a secondary analysis, leaving 148 patients with stage I-III disease in the primary cohort (Figure 1).

This retrospective analysis was conducted within the framework of the institutional protocol entitled ‘Establishment of the Lung Cancer Comprehensive Treatment Biobank at Jinshan Hospital, Fudan University’ (protocol version 1.0, 10 July 2025). The protocol underwent expedited review and received final approval from the Ethics Committee of Jinshan Hospital, Fudan University (approval No. JIEC 2025-S60; final approval date: 29 August 2025). The requirement for individual informed consent for the retrospective use of anonymized clinical data was waived. Medical-record extraction and research analyses were conducted after final approval.

### 2.2. Exposure and Covariates

The primary exposure was pre-treatment NLR, calculated as the absolute neutrophil count divided by the absolute lymphocyte count. NLR was analyzed as a continuous variable. The complete blood count used to calculate NLR was obtained within 7 days before the index treatment date; when more than one eligible pre-treatment sample was available, the sample closest to treatment initiation that preceded it was used.

The revised adjustment set comprised age, sex, and clinical stage, chosen to provide clinically important adjustment while limiting model complexity relative to the available number of events. Clinical stage was entered as a categorical variable. Treatment variables were not included because treatment choice can be influenced by stage, performance status, and disease severity and may therefore introduce overadjustment or treatment-selection bias.

### 2.3. Outcome Definition

For patients with stage I–III disease, the primary endpoint was incident distant metastasis documented within 24 months after the index date. All patients in the primary cohort had no evidence of distant metastatic disease at baseline according to the available staging assessment. Outcome status was determined retrospectively from the medical record through a comprehensive clinical assessment. Imaging findings and pathological confirmation, when available, were treated as the principal evidence; medical history, symptoms, physical findings, laboratory and other clinically relevant examination results, and the treating oncologist’s documentation were used as supporting information. Relevant examinations included computed tomography, magnetic resonance imaging, positron emission tomography/computed tomography, bone scintigraphy, or other site-directed imaging when clinically indicated. A positive event required a newly documented distant metastatic lesion or a treating-oncologist diagnosis of distant metastasis that was not present at baseline. Event-free classification required sufficient documentation through the complete 24-month observation window and no evidence of distant metastasis. No blinded central imaging review or uniform retrospective lesion-level RECIST reassessment was performed. Data abstraction was performed by one investigator (Y.W.), and each outcome classification was independently verified by a second reviewer (X.Z.); discrepant classifications were resolved by consensus with a senior oncologist (Q.Z.).

For patients with stage IV disease, the original dataset used a 2-year metastatic status/progression flag based on the same comprehensive review of clinical history, examinations, imaging, and clinician documentation. Because distant metastatic disease was already present at baseline, persistent disease, progression of known lesions, or development of additional metastatic sites does not represent incident metastasis. Stage IV patients were therefore excluded from the primary analysis and included only in a secondary analysis of the original heterogeneous composite endpoint.

### 2.4. Statistical Analysis

Continuous variables are reported as mean (standard deviation), and categorical variables as number (percentage). The primary association was estimated with logistic regression. The unadjusted model included continuous NLR alone; the adjusted model included NLR, age, sex, and categorical clinical stage. The primary analysis used complete data for all model variables and the 24-month outcome; no imputation was performed. With 31 outcome events, no additional covariates were entered, and confidence-interval width was emphasized when interpreting the estimates. All reported *p* values were two-sided, and reporting followed the STROBE statement.

## 3. Results

### 3.1. Analytic Cohort

A total of 527 consecutive unique patients were screened. After hierarchical application of the eligibility criteria, 341 were excluded: 44 lacked pathological confirmation, 58 had initiated treatment elsewhere and had no evaluable untreated baseline, 65 lacked an eligible pre-treatment complete blood count, 71 had incomplete baseline stage or other essential clinical information, and 103 had insufficient 24-month follow-up documentation. The resulting complete-case cohort contained 186 patients. Thirty-eight patients with stage IV disease at baseline were excluded from the primary analysis, leaving 148 patients with stage I–III disease. Within 24 months, 31 patients (20.9%) had incident distant metastasis and 117 remained event-free (Figure 1). Baseline characteristics of the primary cohort are shown in Table 1.

### 3.2. Association Between NLR and Incident Distant Metastasis

Mean pre-treatment NLR was 2.83 (SD 0.79) among patients who developed distant metastasis and 2.07 (SD 0.85) among those who did not. Each one-unit increase in NLR was associated with higher odds of distant metastasis in the unadjusted model (OR 2.71, 95% CI 1.65–4.45; *p* < 0.001). The estimate changed little after adjustment for age, sex, and clinical stage (adjusted OR 2.70, 95% CI 1.60–4.57; *p* < 0.001) (Table 2). The number of events limits the precision of more complex models.

### 3.3. Secondary Full-Cohort Analysis

In the full cohort, 49 of 186 patients (26.3%) met the original composite metastatic status/progression endpoint. Events were recorded in 31 of 148 patients with stage I–III disease and 18 of 38 patients with stage IV disease. Continuous NLR was associated with the composite endpoint in an unadjusted model (OR 2.65, 95% CI 1.75–4.01; *p* < 0.001). Because this analysis combines incident metastasis with a different stage IV clinical state, it does not estimate the risk of incident distant metastasis and is presented only in Supplementary Table S1.

## 4. Discussion

In this retrospective cohort, higher pre-treatment NLR was associated with newly documented distant metastasis within 2 years among patients with stage I–III lung cancer. The magnitude of the NLR estimate was similar before and after adjustment for age, sex, and stage. This finding is consistent with reports linking elevated NLR to adverse outcomes in lung cancer [19,20,21,22].

The broader evidence base is now considerable. Higher NLR has been associated with poorer survival across lung cancer treatment settings, including meta-analytic estimates in immunotherapy-treated disease and evaluations in stage III disease [23,24,25,26]. In operable disease, NLR and composite inflammatory scores show prognostic value around surgery and resection [27,28,29,30], and combining NLR with PD-L1 expression adds prognostic information [31,32]. Beyond NLR itself, C-reactive protein, circulating immune indices, and PET-based inflammatory parameters have been associated with outcome and treatment response [33,34,35,36,37], and these inflammatory markers have also been linked to brain metastasis and to early or advanced recurrence [38,39,40,41]. Across this literature, effect sizes and cut points vary, endpoints differ, and most reports are retrospective and single-center; convergence across studies rather than any single estimate therefore supports the present association.

The revised analysis addresses two important sources of bias in the original report. First, patients with stage IV disease were removed from the primary endpoint because persistent or progressive metastatic disease is not equivalent to a new distant metastasis. Second, the original highly adjusted model was replaced with a smaller parsimonious model. With only 31 primary events, even the reduced model remains limited, but it avoids fitting demographic, comorbidity, performance-status, and treatment variables simultaneously. Treatment was excluded because it is partly determined by stage and clinical severity.

The present result should be interpreted as an association, not as a validated prediction tool. NLR was measured once before treatment, and no externally validated cut point was assessed. Analyses of thresholds and small subgroups were removed because the available data could not support stable estimates. The earlier LDH/albumin decomposition was also removed: the biomarkers were measured at the same time, temporal ordering could not be established, and the reported effect scale could not be reconciled reliably with the source analysis.

Several limitations remain. The study was retrospective and single-center, with potential selection bias, residual confounding, and outcome misclassification. Although 527 consecutive unique patients were screened, 341 were excluded under hierarchical criteria, including 103 with insufficient 24-month outcome documentation; this complete-case selection may limit generalizability. Outcome classification reflected routine clinical practice and comprehensive record review rather than blinded central imaging review or uniform lesion-level RECIST reassessment. Follow-up intensity and the choice of diagnostic examinations may have varied between patients. Acute inflammatory conditions, medication exposure, and other factors capable of altering peripheral leukocyte counts could not be fully standardized. The primary analysis included only 31 events, and the adjusted model therefore has limited precision. The secondary full-cohort endpoint remains clinically heterogeneous, and the findings have not been externally validated. Patients with insufficient follow-up may differ systematically from those retained (for example, by having more advanced or more indolent disease, poorer access to surveillance imaging, or competing causes of early mortality), so the cohort is likely skewed toward patients with more complete care, and the observed association may not be transportable to settings with different follow-up intensity or patient mix.

NLR is inexpensive and routinely available, but its clinical value should be tested in prospective multicenter cohorts with prespecified imaging and endpoint adjudication, adequate event numbers, repeated biomarker measurements, and independent validation.

## 5. Conclusions

Higher pre-treatment NLR was associated with 2-year incident distant metastasis among patients with stage I–III lung cancer in this single-center retrospective cohort. The finding is hypothesis-generating and requires prospective external validation. It does not establish causality or support a clinically actionable NLR threshold.

## Figures and Tables

**Table 1:** Baseline characteristics of the stage I–III primary cohort.

Characteristic	Total (*n* = 148)	No Distant Metastasis (*n* = 117)	Distant Metastasis (*n* = 31)
Age, years	63.8 (9.3)	63.5 (9.4)	65.0 (8.9)
Sex, *n* (%)			
Female	63 (42.6)	46 (39.3)	17 (54.8)
Male	85 (57.4)	71 (60.7)	14 (45.2)
Clinical stage, *n* (%)			
I	43 (29.1)	39 (33.3)	4 (12.9)
II	44 (29.7)	37 (31.6)	7 (22.6)
III	61 (41.2)	41 (35.0)	20 (64.5)
BMI, kg/m^2^	24.0 (3.5)	23.9 (3.4)	24.7 (3.6)
Smoking status, *n* (%)			
Current	28 (18.9)	20 (17.1)	8 (25.8)
Former	35 (23.6)	25 (21.4)	10 (32.3)
Never	85 (57.4)	72 (61.5)	13 (41.9)
Histology, *n* (%)			
Adenocarcinoma	83 (56.1)	64 (54.7)	19 (61.3)
Squamous	44 (29.7)	33 (28.2)	11 (35.5)
Other	21 (14.2)	20 (17.1)	1 (3.2)
ECOG performance status, *n* (%)			
0	70 (47.3)	57 (48.7)	13 (41.9)
1	52 (35.1)	40 (34.2)	12 (38.7)
2	22 (14.9)	17 (14.5)	5 (16.1)
3	4 (2.7)	3 (2.6)	1 (3.2)
Pre-treatment NLR	2.23 (0.89)	2.07 (0.85)	2.83 (0.79)

Values are mean (standard deviation) or *n* (%). BMI, body mass index; ECOG, Eastern Cooperative Oncology Group; NLR, neutrophil-to-lymphocyte ratio.

**Table 2:** Association between pre-treatment NLR and 2-year incident distant metastasis in the stage I–III cohort.

Model	*n*	Events	OR per 1-Unit NLR (95% CI)	*p*-Value
Unadjusted	148	31	2.71 (1.65–4.45)	<0.001
Adjusted for age, sex, and clinical stage	148	31	2.70 (1.60–4.57)	<0.001

Clinical stage was modeled categorically. CI, confidence interval; NLR, neutrophil-to-lymphocyte ratio; OR, odds ratio.

## Data Availability

The patient-level data are not publicly available because they contain hospital-derived clinical information subject to institutional privacy and ethics restrictions. De-identified data may be available from the corresponding author on reasonable request and with institutional approval.

## Supplementary Materials

The following supporting information can be downloaded at: https://www.mdpi.com/article/10.3390/jcm15166207/s1, Table S1: Secondary full-cohort analysis of the original composite metastatic status/progression endpoint.
