# Supplementary material for: Baseline Neutrophil-to-Lymphocyte Ratio and Two-Year Incident Distant Metastasis in Stage I–III Lung Cancer: A Single-Center Retrospective Cohort Study
Source: J Clin Med. 2026 Aug 11;15(16):6207. doi: 10.3390/jcm15166207 (PMC13513032; doi:10.3390/jcm15166207)
Supplement: Supplementary file 1 [file jcm-15-06207-s001.zip › jcm-4417043-supplementary.pdf]

Supplementary Table S1. Secondary full-cohort analysis of the original composite metastatic status/progression endpoint

Population / analysis	n	Events	Event rate	Estimate
Overall cohort	186	49	26.3%	-
Stage I	43	4	9.3%	-
Stage II	44	7	15.9%	-
Stage III	61	20	32.8%	-
Stage IV	38	18	47.4%	-
Continuous NLR, unadjusted	186	49	-	OR 2.65 (95% CI 1.75-4.01); p<0.001
